# Supplementary material for: Double-layered protein nanoparticles induce broad protection against divergent influenza A viruses
Source: Nat Commun. 2018 Jan 24;9:359. doi: 10.1038/s41467-017-02725-4 (PMC5783933; doi:10.1038/s41467-017-02725-4)
Supplement: Supplementary file 1 — Supplementary Information [file 41467_2017_2725_MOESM1_ESM.pdf]

Supplementary Information

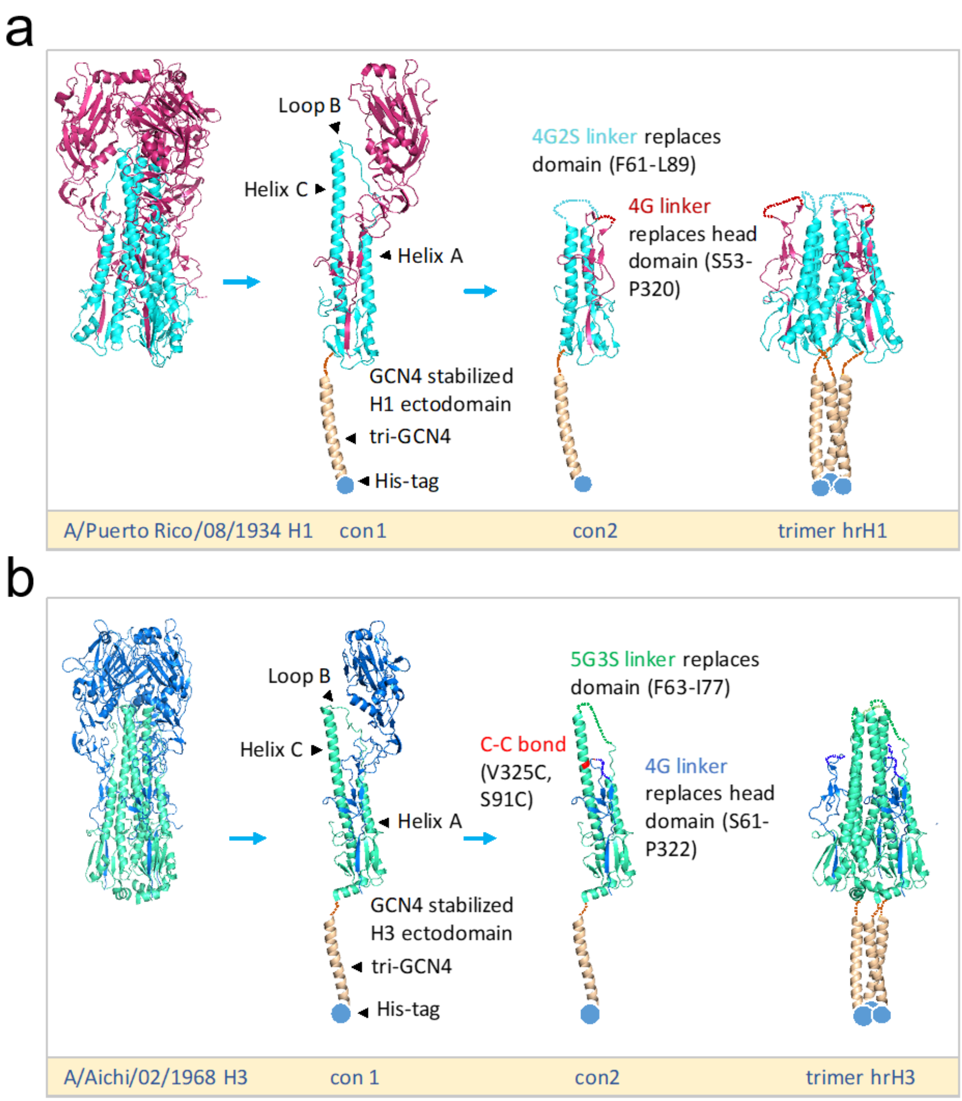

Supplementary Fig. 1. Schematic diagram of structure-based hrHA construction. **a**, Cartoon models of hrH1 generation. Structure-based deletion of HA head domain was diagramed in the model of PR8 H1 (HA, PDB: 1rvx). The HA1 polypeptide is colored in magenta, HA2 in cyan, and modified trimerization motif GCN4 (Tri-GCN4) in wheat color. **b**, Cartoon models of hrH3 design. Optimization started with the ectodomain of Aic H3 (PDB: 3ztj). HA1 polypeptide is colored in blue, HA2 in green-cyan, and modified trimerization motif GCN4 in wheat. Dash lines indicate linkers. The images were generated using PyMOL and Microsoft PowerPoint software.

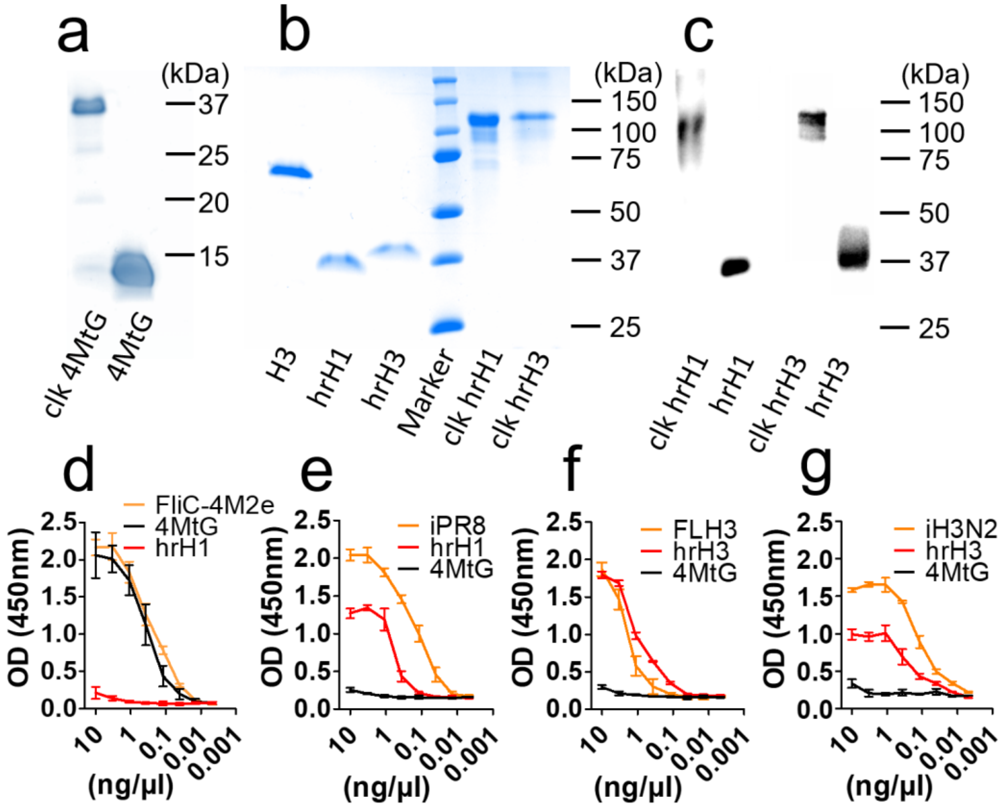

Supplementary Fig. 2. Characterization of recombinant proteins. a, 4MtG; **b**, hrH1 and hrH3. SDS-PAGE was carried out under reducing condition. **c**, Crosslinked (clk) and non-crosslinked hrH1 and hrH3 was analyzed in reducing SDS-PAGE and Western Blot using anti-His monoclonal antibody. **d-g**, Sandwich ELISA of 4MtG, hrH1 and hrH3 using capture antibodies 14C2 (**d**), C179 (**e**), 12D1 (**f**) and 9H10 (**g**), respectively. FliC-4M2e (**d**), inactivated PR8 H1N1 (iPR8) (**e**), FLH3 protein (**f**) and inactivated Aic H3N2 (iH3N2) (**g**) are positive controls. The hrH1 (**d**) and recombinant 4MtG (**e-g**) are negative controls. Data are presented as mean ± s.d. (n=3). The experiments were repeated twice with similar results.

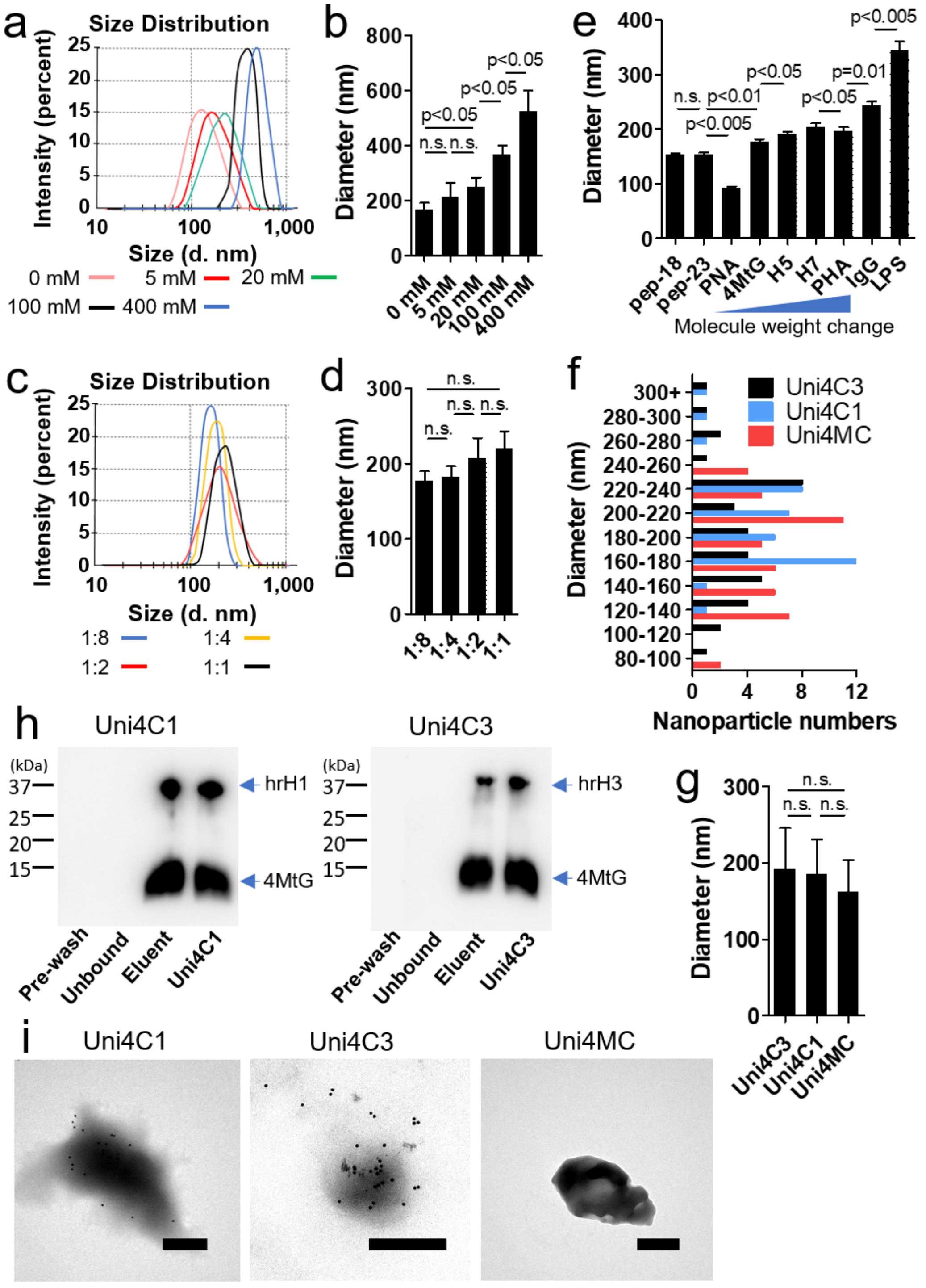

Supplementary Fig. 3. PNp characterization and optimization of PNp fabrication. a, Size distribution of PNps. PNps were fabricated under different conditions with varying amounts of DTSSP crosslinker. The distribution of PNp hydrodynamic diameters was measured in purified water by dynamic light scattering (DLS) and plotted as a function of the intensity of the signal for a given size as a percentage of the total signal intensity. **b**, Bar chart depicting the size variation of desolvated Uni4MC with increasing amounts

of DTSSP. **c**, Size distribution of PNps desolvated under different conditions with varying desolvation ratios of protein solution to absolute ethanol. **d**, Bar chart depicting the size variation of desolvated Uni4MC with different ratios of sample/absolute ethanol volume. **e**, Bar chart depicting PNp size variation with different materials including peptides containing 18 a.a. (pep-18) or 23 a.a. (pep-23), peanut agglutinin (PNA), 4MtG, H5, H7, Phytohaemagglutinin (PHA), IgG and lipopolysaccharide (LPS). PNps were fabricated in 4 volumes of absolute ethanol and in the absence of a crosslinker. The Blue bar indicates the increasing molecule weight change (n=3). **f**, Analysis of the size distribution of Uni4C3, Uni4C1 and Uni4MC in SEM images using ImageQuantTL software. **g**, Comparison of protein nanoparticle diameters of Uni4C3, Uni4C1 and Uni4MC. **h**, Western Blot analysis of Pre-wash, Unbound fraction and Eluent samples in pull-down assay. Uni4C1 and Uni4C3 were used as positive controls. **i**, Images of immuno-gold stained Uni4C1, Uni4C3 and Uni4MC using transmission electronic microscopy. C179, 12D1 and a mix of these two monoclonal antibodies were used as the primary antibodies in staining Uni4C1, Uni4C3 and Uni4MC, respectively (Bars represent 200 nm in length). Data are presented as mean ± s.d. Statistical significance was analyzed by t-test for **b**, **e**, **d** and **g**. P values shown in bar charts (n=3). n.s. indicates no significance between two compared groups. The experiments were repeated twice with similar results.

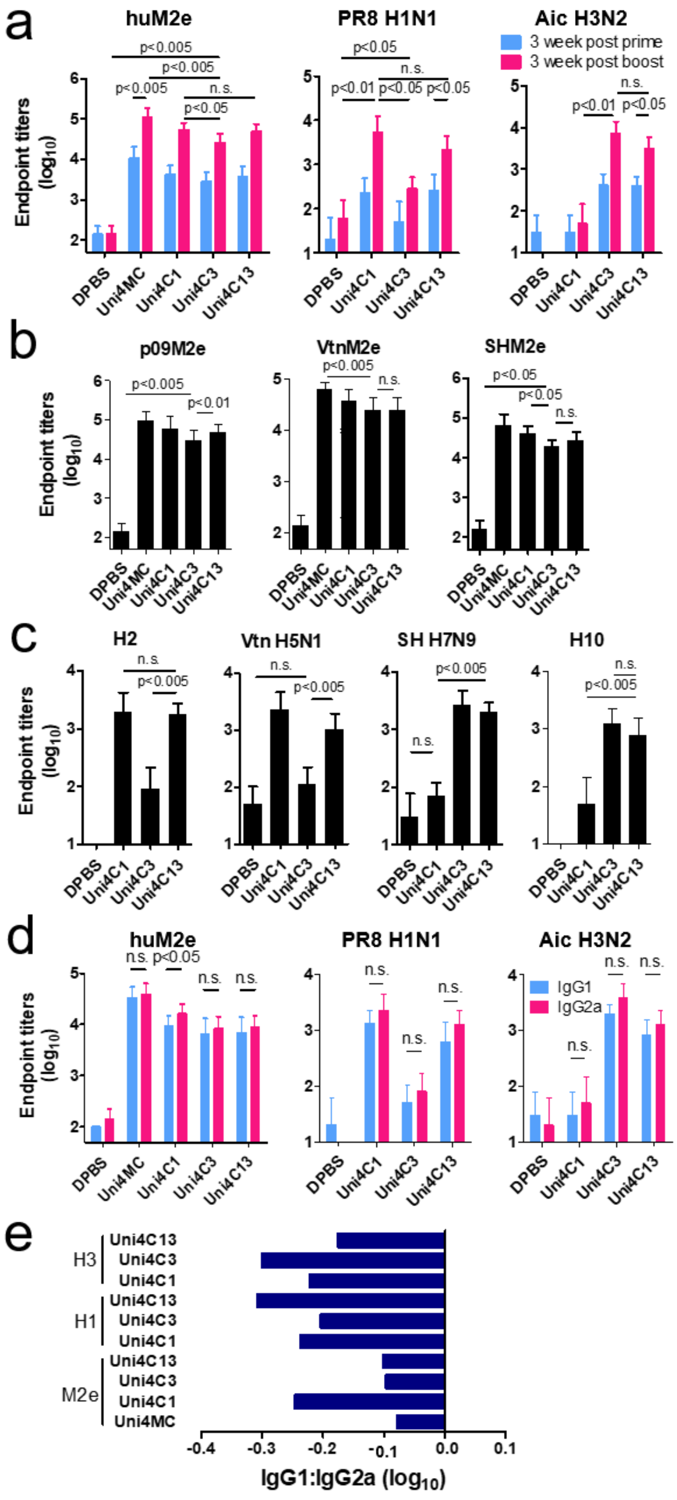

Supplementary Fig. 4. Humoral immune responses of immunized mice. a, IgG titers. IgG titers against 4MtG, formalin-inactivated PR8 H1N1 and formalin-inactivated Aic H3N2 were determined with pre-immune sera and serum samples collected 3 weeks post each immunization. **b**, M2e binding spectrum. Serum IgG binding capacity

to various M2e peptides, including p09M2e, VtnM2e and SHM2e were evaluated. **c**, Binding activity to various HAs. Serum IgG binding to different HAs, including H2 and H10 from FL HA-transfected HEK293T cells and H5 and H7 from formalin-inactivated viruses, were compared. **d**, Determination of IgG1 and IgG2a titers against M2e, formalin-inactivated PR8 H1N1 and formalin-inactivated Aic H3N2 using ELISA. **e**, Bar chart showing logarithm values of the ratio IgG1:IgG2a. Data are presented as mean ± s.d. (n=10). Statistical significance was analyzed by t-test for **a-d**. P values shown in bar charts and n.s. indicates no significance between two compared groups. The experiments were repeated twice with similar results.

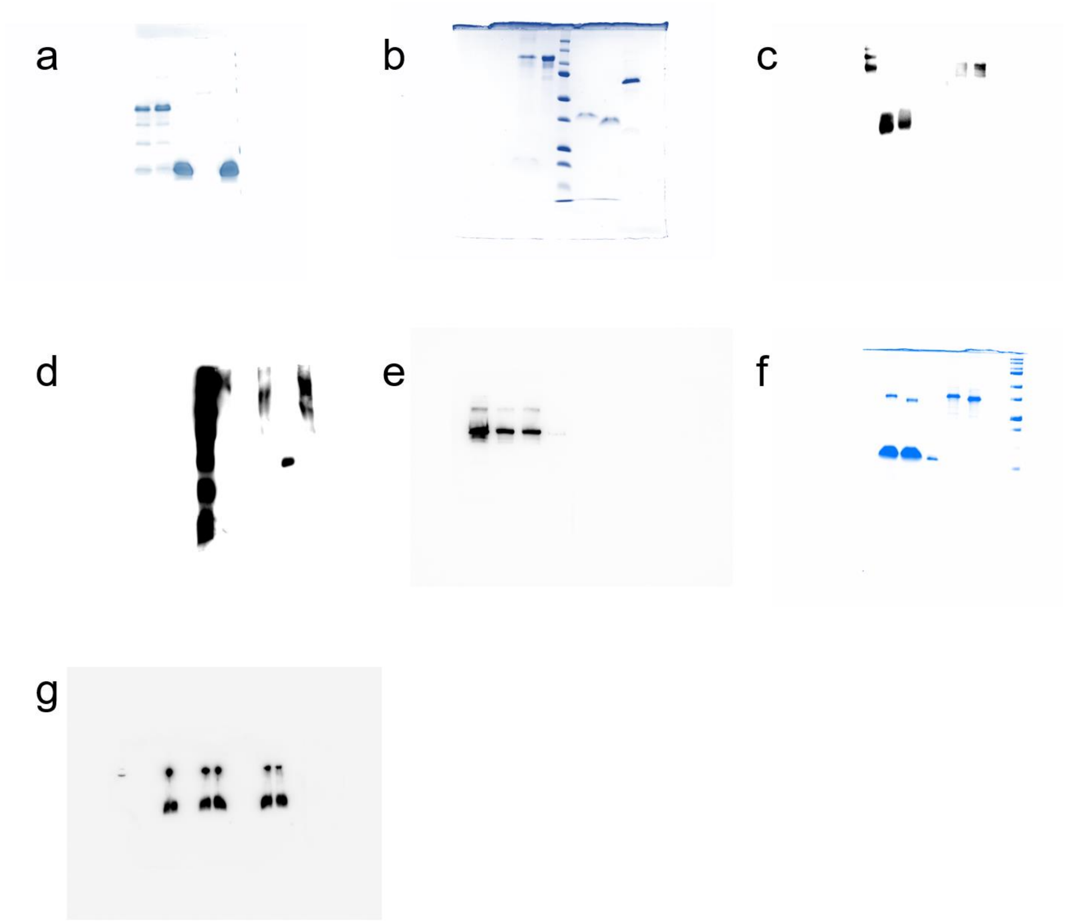

Supplementary Fig. 5. Uncropped scanned western blots and Coomassie blue stained SDS-PAGE gels. a, SDS-PAGE gel analysis of BS3-crosslinked 4MtG. SDS-PAGE gel analysis (**b**) and western blot analysis (**c, d**) of BS3-crosslinked hrH1 and hrH3. **e**, Western blot analysis of Uni4MC. **f**, SDS-PAGE gel analysis of Uni4C1 and Uni4C3. **g**, Western blot analysis of samples from pull-down assay.

Supplementary Table 1. Various M2e sequences*

Codes	Sequence	Origin
**PR8M2e**	SLLTEVETPIRNEWGCRCNGSSD	A/Puerto Rico/8/1934 (H1N1)
**AicM2e**	SLLTEVETPIRNEWGCRCNDSSD (huM2e)	A/Aichi/2/1968 (H3N2)
**p09M2e**	SLLTEVETPTRSEWECRCSDSSD	A/California/7/2009 (H1N1)
**PhiM2e**	SLLTEVETPIRNEWGCRCNDSSD (huM2e)	A/Philippines/2/1982 (H3N2)
**rVnM2e**	SLLTEVETPIRNEWGCRCNGSSD	A/Vietnam/1203/2004 (H5N1)
**rSHM2e**	SLLTEVETPIRNEWGCRCNGSSD	A/Shanghai/2/2013 (H7N9)
**4MtG**	SLLTEVETPIRNEWGSRSNDSSD PGGSSGGSS SLLTEVETPTRSEWESRSSDSSD PGGSSGGSS<br>Human consensus Linker Swine consensus Linker<br>SLLTEVETPTRNGWESKSSGSSD PGSGSGSGS SLLTEVETPTRNGWESNSSDSSD PGGGGSSSS<br>Avian consensus Linker Domestic fowl consensus Linker<br>LELKQIEDKLEEILSKLYHIENELARIKKLLGE·<br>Tetra-GCN4	

*Amino acid residues in orange represent differences from the human viral M2e consensus (huM2e). All cysteine (C) residues in consensus M2e sequences in 4MtG construct were mutated into serine (S). Sequences highlighted with yellow in 4MtG are flexible linkers. The sequence in blue is the tetramerization sequence tetra-GCN4.

Supplementary Note 1. Nucleotide sequence of 4MtG.

ATG AAG TTC CTG GTC AAC GTC GCC CTC GTC TTT ATG GTG GTT TAT ATT
AGC TAT ATA TAC GCC GAT TCG CTT CTC ACC GAG GTG GAG ACG CCT ATA
AGA AAT GAA TGG GGT TCA AGA TCT AAT GAT TCG TCC GAT CCG GGT GGT
TCT TCG GGT GGC AGT AGT TCG CTG CTC ACA GAA GTG GAA ACC CCT ACT
CGC TCC GAA TGG GAA TCC CGC TCA TCT GAC TCA TCA GAT CCG GGA GGC
TCC AGC GGC GGC TCG TCA TCC CTT CTT ACA GAA GTA GAA ACT CCA ACT
AGG AAT GGA TGG GAG TCG AAG AGC AGT GGC TCC TCT GAC CCC GGT TCT
GGC TCC GGT AGT GGC AGT TCT TTG CTG ACG GAG GTG GAA ACT CCA ACC
AGG AAC GGC TGG GAA AGC AAC TCC TCC GAC AGT TCT GAC CCA GGC GGC
GGC GGT AGC AGT TCC TCG CTT GAG CTG AAG CAA ATT GAG GAT AAA CTG
GAA GAG ATT CTG AGT AAG CTC TAC CAT ATC GAA AAC GAA CTG GCC AGA
ATC AAG AAA TTG CTC GGA GAG CAT CAC CAC CAT CAT CAT TGA
